# Value dynamics and interpersonal tension among astronauts during International Space Station missions

**DOI:** 10.1371/journal.pone.0351965

**Published:** 2026-07-08

**Authors:** Gro Mjeldheim Sandal, Nathan Smith

**Affiliations:** 1 Department of Psychosocial Science, University of Bergen, Bergen, Norway; 2 Centre for Peace and Security, Coventry University, Coventry, United Kingdom; Naval Health Research Center, UNITED STATES OF AMERICA

## Abstract

Astronauts’ ability to maintain motivation over extended periods is crucial for space mission success. This paper examines how motivational goals (personal values) change during space missions and explores the associations between perceived value congruence and intra-crew tension among astronauts staying 4–7 months at the International Space Station. Twelve astronauts regularly completed the Crew Values Questionnaire (CVQ) package, assessing personal values, perceived value congruence of crew members, and tension attributed to perceived value incongruence. Overall, they rated their values, particularly universalism, benevolence, and tradition, as highly concordant. Temporal analyses showed that scores for hedonism and power increased early in missions and declined later, while benevolence and security decreased and rose again towards the end and into post-mission. Perceived value-congruence followed similar trajectories, with differences in achievement, power, and benevolence decreasing and then increasing across mission phases. Multilevel modeling showed that perceived incongruence in seven out of eight personal values significantly predicted interpersonal tension. In conclusion, in-mission adjustments of value priorities may have helped astronauts sustain motivation, but these shifts could also influence crew dynamics. Pre-mission training and in-flight support should target shifts in motivational sources and manage interpersonal tensions from value diversity to prevent adverse outcomes and leverage crew heterogeneity.

## Introduction

During long-duration ‌‌human space missions, astronauts live and work under extreme conditions that create risks for safety, performance, and health. A particular challenge pertinent to extended duration missions is hypo-stimulation induced by monotony, repetitiveness, and isolation. Hypo-stimulation can substantially affect brain structures and contribute to sleep disruptions, impaired cognitive performance, negative affect, and deteriorations in motivation and crew cohesion [1]. Such reactions not only threaten astronauts’ well-being but also lead to general performance deficits and inadequate or delayed responses to off-nominal situations. Consequently, all space agencies now recognize the significance such risks and the need for psychological countermeasures to mitigate of adverse behavioral outcomes and foster resilience and thriving [2]. An essential aspect of in-flight support during extended missions is to enhance motivation to promote optimal activation levels, which can indirectly influence individual adaptation and team relationships. One way to sustain motivation is by developing and offering individually tailored leisure activities that accommodate astronauts’ evolving interests and needs throughout the mission [3,4]. Despite its importance, there remains little empirical knowledge about the dynamics of astronauts’ motivation during space missions.

The present paper stems from a unique opportunity to monitor astronauts’ motivation and crew relationships during long-duration missions at the International Space Station (ISS), building on space simulation studies that took place as part of the Mars 500 project [5,6] and the Chinese lunar project [7]. The work aims to contribute empirical evidence related to potential alterations in astronauts’ motivational goals throughout the course of their missions and explores how diversity in such’ motivational goals may be associated with intra-crew tension. Team performance closely associated with individual adaptation, is essential for mission success and is expected to become even more critical in future exploratory missions, where crews will operate with greater autonomy than in Low Earth Orbit (LEO). Pre-flight and potentially in-flight assessments of team compatibility based on individual characteristics, such as motivational goal priorities, could serve as a valuable countermeasure, potentially leading to increased crew resilience in managing mission stressors [8].

We draw upon the comprehensive taxonomy of personal values proposed by Schwartz and Rubel [9], who conceptualized values as broad motivational trans-situational goals of varying importance that guide attention and action to intrinsically rewarding social, intellectual, and emotional opportunities. From this perspective, fulfillment of personal values is integral and essential to well-being [10]. Personal values are hierarchically organized and represented in different goal prioritizations [10]. For example, emphasis on power tends to trigger salient goals that allow individuals to influence or exert control over a situation [11] while emphasis on benevolence is associated with a team orientation reflecting care and interest in others [12]. Research founded in the person-environment fit framework [13] has shown that congruence in values promotes interpersonal attraction, enhances communication, and creates trust within organizations [14], and is therefore known for being an important influence of cooperative behavior and efficiency in teams [15,16]. In this study we presumed that differences in goal prioritization might pose significant challenges for interdependent work teams operating under challenging and safety critical conditions, such as space missions – potentially leading to conflicts, loss of crew cohesion, and sub-optimal performance.

### Alterations in values during space missions

In the first part of this study, we examined potential alterations in personal values among the astronauts over long-duration ISS-missions. Although personal values are generally considered relatively stable and durable throughout the life span [17–20] they can be susceptible to change when adapting to new environments and life transitions or in response to profound and challenging experiences [18,21]. Space missions might serve as a prime example of such experiences due to the extreme nature of having to adjust to living and working in this context. Indicating such effects, text analysis on archival materials observed changes in astronauts’ references to personal values from pre- to post space missions [22,23]. Notably, emphasis on universalism, a value referring to equality and harmony with nature, was mentioned during and after the flight by many astronauts [23]. Another study, in which retired astronauts were asked to reflect on their careers, found that many recalled experiencing positive personal growths after their spaceflights, including more appreciation of life and an increase in reference to power and self-direction [24]. The increase in power and self-direction reflecting more emphasis on social recognition and autonomy. Whilst this work has been influential and informative, the reliance on autobiographical material and retrospective memories provide a limited understanding of how the personal values of astronauts in space might change over time. Other scholars have utilized standardized questionnaires to assess values among personnel spending time in isolated and confined environments considered Earth-based analogues to human space missions, such as experimental simulation studies and polar expeditions [5–7,25].

Building on previous research, the present study offers a unique and more comprehensive perspective by examining prospective temporal short-term changes in the personal values of astronauts as they progress through various phases of space missions, each marked by their own physical, psychological, and interpersonal challenges. Quantitative analysis of astronaut journals revealed that the emotional valence of potential stressors tends to differ according to the time of ISS missions. For example, personal space was considered more important in the third mission quarter [26], indicating shifts in the significance attached to certain living conditions as the mission develops. These observations underscore the dynamic nature of motivational goals in response to evolving environmental and psychological demands.

Bardi and Goodwill [27] describe two main roads to value change. The first involves changes prompted by environmental cues that automatically prime or raise awareness of certain values. Through this mechanism, frustrations linked to deficit needs, referring to basic physical and emotional requirements, may lead to shifts in value priorities [28]. This aligns with observations during space simulation studies. A decreased emphasis on benevolence occurred simultaneously as crew members exhibited reduced interest in group activities and an increased need for privacy [6]. An increased emphasis on hedonism coincided with expressions of strong dissatisfaction regarding the quality and type of food provided [5]. These findings showing that value changes were meaningfully related to mission events, are consistent with tenets of the Self-Determination Theory [29], which highlights the importance of physical and social context and the interconnectedness of values, needs, and emotions [30,31].

Secondly, and more commonly, individuals tend to adjust their value priorities in response to environmental contingencies by de-emphasizing or downgrading values that are difficult or impossible to pursue in the current situation [27]. According to Lazarus and Folkman’s [32] transactional stress theory, challenging situations trigger a dynamic process involving continuous appraisal, coping strategies, and emotional responses. Similarly, Bonanno and Burton [33] highlight that a crucial factor for psychological resilience under stress is flexible self-regulation, being able to adapt one’s thoughts, feelings, and behaviours as needed. Based on these perspectives, the active adjustments of value priorities may help astronauts better regulate their internal states and actions, preventing them from drifting away from their long-term goals [34]. This adaptive process can then support sustained performance and well-being throughout the entire mission.

In summary, theories and findings from previous space research and ground-based simulation studies indicate that space crew members are likely to experience changes in their personal values and motivation during long-duration missions. These alterations may be driven by different underlying mechanisms. Gaining a better understanding of these changes is essential for space agencies to develop effective countermeasures that address the psychological challenges specific to each phase of the mission. Although empirical evidence from simulations provides some initial insights, there is a notable lack of research monitoring personal values and their changes among astronauts during actual space missions.

### Personal values and crew tension

The second part of this study addressed the association between diversity in personal values and crew dynamics, focusing on interpersonal tension as the primary outcome. Crew cohesion is crucial for mission success, especially on long-duration missions, where complex goals require a strong team to maintain relations and work together effectively [35]. Interpersonal tension, a common consequence of isolation and confinement [36,37], can significantly impair crew communication and coordination, with potentially serious safety and performance-related implications. Although anecdotal situations with crew tension from space have been reported, astronauts often avoid expressing overt aggression [37,38]. Tension can still generate negative emotions that distract from shared goals, impair performance, and compromise safety. A better understanding of how diversity in individual characteristics among crew members might impact team functions, positively or negatively, has been called for [39]. These are elements that can be targeted during crew composition and training. To this end, space psychologists have recommended that a constellation of crew members should be avoided whose values, needs and beliefs are competitive or incongruent [5]. However, the implications of diversity in personal values for group dynamics during actual space operations remain largely unexplored.

International space crews consist of members from different organizational, professional and national cultures, each of which may impact on their personal values. Research has observed variations among national space agencies in critical operational aspects such as leadership styles, adherence to procedures, and collectivistic orientations. [40–42]. However, even astronauts with similar cultural backgrounds can differ in personal value profiles. Research suggests that such individual variations may lead to differences in how crew members prioritize, interpret, and respond to stimuli, increasing the likelihood of tension and poor performance outcomes [39,43]. Studies examining group dynamics in confined settings have observed clique formation [5,6,44,45], which in several instances seemed linked to perceived value congruence. Individuals deviating from the group have become socially isolated [46–48]. These findings align with social psychological theories emphasizing the importance of perceived similarity for interpersonal attraction and group cohesion [49,50]. Although subgrouping is not inherently negative, there is strong evidence that clique formation can foster conflicts between in-group and out-group members and escalate more tension [36]. Indeed, culturally related value incongruence were assumed to play an important role in a severe conflict between crews living in different modules during an ISS simulation [45]. Also, anecdotally, tension attributed to culturally ingrained attitudes and behaviours, was reported since the early Russian space missions involving foreign astronauts [47,48].

Overall, theories and evidence suggest that diversity in personal values can have a profound impact on team dynamics in space. However, existing research is limited to traditional work teams, anecdotal reports, and space simulation studies, which may not fully generalize to real missions in space [51]. Space crews are highly selected and have undergone extensive training programs and thus might be able to deal with interpersonal challenges differently from other teams. This paper makes an important contribution to the literature by analysing data from actual space operations, providing insights into how perceived value congruence might impact crew dynamics across the phases of space missions**.** When studying value congruence through a temporal lens, we recognize that changes in perceived congruence may occur due to actual alterations in the astronauts’ personal values, stemming from the two mechanisms outlined earlier, as well as from variations in interpretative or even heuristic processes. In this work, we build on an accumulating body of evidence within organizational research which has challenged the commonly held assumption that value congruence represents a stable construct [52,53], and test the proposition that individuals’ perceptions of value congruence are, in fact, dynamic [54,55]. From this perspective, we can hypothesise that perceptions of value congruence are likely to fluctuate because they are embedded in individuals’ ongoing personal (e.g., social relationships) and impersonal (e.g., procedures) interactions with the environment. Individual differences in perceived value congruence can in turn have important consequences for an individual’s thoughts, feelings, and behaviour in the workplace, which, considered in the present case, may have implications for crew functions.

### The current study

The primary goal of this research was to better understand the dynamics of astronaut motivation viewed through the lens of personal values during long-duration missions. To this end, self-report data were collected from astronauts at regular intervals before, during and after ISS missions lasting between four and seven months. Based on the rationale outlined earlier, we expected that value priorities would fluctuate throughout the different phases of the mission. Given the limited and inconsistent findings from previous studies, we did not formulate specific hypotheses regarding the direction or exact nature of these temporal changes. With regard to the secondary aim of this work, drawing from existing theories and team research, we hypothesized that perceived value incongruence (differences in values) between crew members would be associated with intra-crew tension.

## Materials and methods

### Context

In this study we use the terms astronaut to represent any agency-trained and certified individual flying in space. Data was collected between 2013 and 2019 from astronauts during missions aboard the ISS, a space station in low Earth orbit. The space station consists of the Russian Orbital Segment and the United States Orbital Segment shared by many nations. The normally six-person crew typically consists of three Russians, two Americans, and one astronaut from Japan, Canada, or Europe. During the data collection period, about every three months, a Soyuz capsule returned to Earth with three astronauts, and a Soyuz launched from Earth with three astronauts to replace them. The return of a Soyuz to Earth marks the end of an ISS epedition, and at this point the command of the ISS is transferred to another crew member. The crew members spend most of their time on the ISS performing experiments and maintenance, and at least two hours daily are allocated to exercise and personal care. Mission control centres in Houston and Moscow assist the crew aboard the ISS.

### Sample

Twelve male astronauts from ten increments participated in this study. An increment refers to the time between crew exchanges on the ISS. The astronauts spent between 168 and 215 days at the space station. Age ranged from 40 to 54 years (M = 45,5 SD = 4,15). Three participants served as commanders in parts of their missions. Among the participants, two were on their first spaceflight, seven had completed their second, two had their third, and one was participating in his sixth mission. Additional details on mission increment and astronaut characteristics, including agency, are withheld to preserve subject confidentiality and anonymity.

### Instrument

The Crew Values Questionnaires (CVQ) package was used to monitor the astronauts’ personal values and their perceptions of value-related differences and value-related crew tension. PCVQ consists of three parts. Part 1 includes the 21-item version of the Portrait Values Questionnaire (PVQ) [56,57] and comprises short verbal portraits of different people, gender-matched to the respondent. Each portrait describes a person’s goals, aspirations, or wishes, which point implicitly to a personal value. For example: “It is important to him to make his own decisions about what he does. He likes to be free to plan and not depend on others” describes a person for whom self-direction values are essential. For each portrait, the respondent answers the question “How much like you is this person?” on a six-point scale from “very much like me (6)” to “not like me at all (1)”. Personal values are inferred from self-reported similarity to the person portrait. An extensive body of studies in numerous countries has demonstrated the reliability and validity of the PVQ [57]. Table 1 provides definitions of each value construct in terms of its central goal.

**Table 1:** Definitions of personal values [56,57].

Value type	Defining motivational goal
**Self-direction**	Independent thought and action-choosing, creating, exploring
**Stimulation**	Excitement, novelty, and challenge in life
**Hedonism**	Pleasure or sensuous gratification for oneself
**Achievement**	Personal success through demonstrating competence according to social standards.
**Power**	Social status and prestige, control or dominance over people and resources
**Security**	Safety, harmony, and stability of society, of relationships, and of self
**Conformity**	Restraint of actions, inclinations, and impulses likely to upset or harm others and violate social expectations or norms
**Tradition**	Respect, commitment, and acceptance of the customs and ideas that one’s culture or religion provides.
**Benevolence**	Preserving and enhancing the welfare of those with whom one is in frequent personal contact (the ‘in-group’).
**Universalism**	Understanding, appreciation, tolerance, and protection for the welfare of all people and for nature.

Parts 2 and 3 (5, 6)have been developed and previously used in space simulation studies. In part 2, the participant indicated how much they felt that the members of their crew differed from each other on each value. In part 3, participants indicated how much they considered that differences between crew members in each value contributed to interpersonal tension within the crew. Responses were given on five-point scales from 1(not at all) to 5 (very much). By error, items related to universalism and security were not included in parts 2 and 3 of the survey pack.

### Procedure

The PCVQ was answered in the native language of the astronauts once prior to the mission (- 60 days), every 30 days (-/ + 9 days) in-flight, and once post-mission (+7–10 days). The first in-flight session took place in the third mission week. The questionnaires were administered via on-board computers. After completing the questionnaires, the associated files were saved on PCMCIA-cards and protected with individual passwords and returned to Earth by a Cargo ship (DON17).

### Ethics

Procedures for data collection were reviewed and approved every second year by ESA Medical Board and the Human Research Multilateral Review Board (HMRBR, protocol number 13/012). The project was considered by the Regional Committee for Medical and Health Research Ethics in Norway (ref. 2012/2013) but was exempted for the review as it did not gather sensitive health information. Prior to the start of the study, all subjects received oral and written information in their native language. A “layman description” was provided to ensure that participants understood the nature of the project and potential risks. Participants signed the Multinational Space Station Human Research Informed Consent (Form 1418) before participation.

### Data analysis

After cleaning and screening the data, we computed mean (M) and standard deviation (SD) scores for the questionnaire subscales. Consistent with suggestions by Schwartz [56] individual personal values were centered to control for individual response tendencies and to allow comparison across participants. Positively scored values are interpreted as being higher (i.e., more concordant) in the hierarchy, and negatively scored values are interpreted as lower (i.e., less concordant).

For our main analyses, we first graphed astronauts’ self-assessments of their personal values across the pre- and post-mission phases. This allowed us to visualise the personal value hierarchies and any changes that occurred pre- to post-flight. Next, we used multilevel models to test linear and quadratic temporal changes in self-assessed personal values, perceived value-related differences and perceived value-related tension across the pre-, during-, and post mission phases. Multi-level analytical approaches have previously been used to examine similar types of temporal data derived from small groups in space and expedition contexts [58,59]. Such modelling is also particularly robust to uneven nesting, which is important in the present work where we have different numbers of responses within individuals due to both missing survey responses and slightly different mission durations [60]. To run the multi-level analysis, we developed a multilevel model for each dependent variable (i.e., each personal value, value-related difference and value-related tension variable). Initially, we partitioned variance associated with data nesting by computing empty models that included only a random intercept and constant as a predictor for each dependent variable. Afterwards, we added a fixed effect time (1–9) and squared (1–81) variable to these empty models to allow us to test for linear and quadratic effects. In the final part of our analysis we examined whether perceived value-related differences were related to perceived value-related tension. Again, we used multilevel models to do this. Consistent with the previous approach, we produced empty models for each dependent variable (i.e., each value-related tension variable). We then individually added the associated value-related difference variable into each of the models as a fixed effect. All multilevel analyses were conducted using JASP 0.19.3. The syntax for the analyses is available in the supplementary material.

### Use of Artificial Intelligence

A chatbot-based AI (Microsoft Copilot) was employed solely to improve the clarity and readability of a small number of sentences (5–10). The authors reviewed all AI-suggested edits carefully, verified their accuracy, and ensured that no content, interpretations, or data analyses were generated by AI. All conceptual, methodological, analytical, and interpretative aspects of the study were developed exclusively by the authors.

## Results

### Values pre-and post-mission

**Fig 1:**
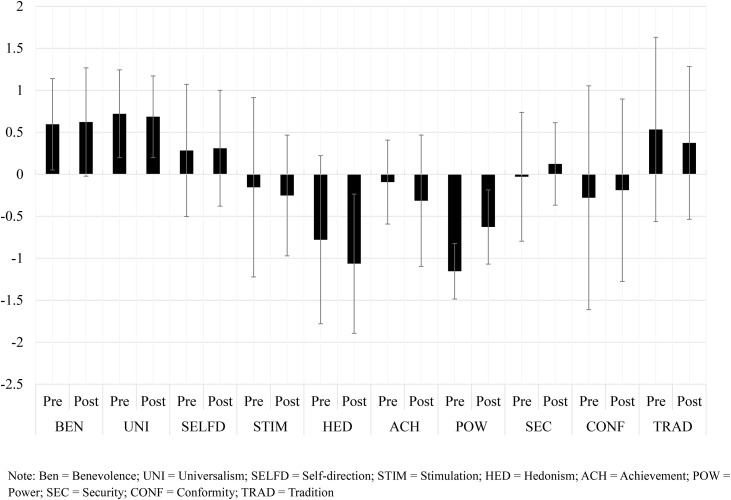
Pre- and post-flight standardized scores across value domains (mean ± SD). Note: Ben = Benevolence; UNI = Universalism; SELFD = Self-direction; STIM = Stimulation; HED = Hedonism; ACH = Achievement; POW = Power; SEC = Security; CONF = Conformity; TRAD = Tradition.

Self-assessed personal values pre-and post-mission are reported in Fig 1. Astronauts rated universalism as their most concordant value followed by benevolence and tradition (see Fig 1). Power and hedonism were the least concordant values. At the overall level, findings suggest high rank order stability in personal values with minor differences between the pre- and post-mission assessments. One notable exception is for power, which was rated as being more concordant post-mission. Power moved from the bottom of the astronauts’ value hierarchy and was replaced by hedonism as the least concordant value.

### Temporal changes in personal values

Table 2 contains the linear and quadratic temporal effects for self-assessed personal values across the pre-, during- and post-phases of the missions. There were positive linear and negative quadratic effects for both hedonism and power. This suggests a pattern where scores for these values increased and then declined over the course of the missions. Opposite effects were observed for benevolence. There was a negative linear and positive quadratic effect suggesting that scores for this value declined and then increased later and towards the end of the missions. Although there was only a trend for a negative linear effect, a positive quadratic effect for security suggests a similar pattern to benevolence where scores for this value declined and then increased across the phases of the missions.

**Table 2:** Multi-level model results for time-based changes in personal values.

			Linear		Quadratic	
	*M*	*SD*	*Est*	*t (67)*	*Est*	*t (67)*
**Self-direction**	.47	.62	.05	.60	−.01	−.79
**Stimulation**	−.12	.74	−.06	−.65	.01	.53
**Hedonism**	−.70	.81	.19*	2.27	−.02*	−2.47
**Achievement**	−.31	.62	−.10	−1.19	.01	1.03
**Power**	−.74	.62	.28**	3.58	−.02**	−2.93
**Conformity**	−.19	.92	.09	1.22	−.01	−1.08
**Tradition**	.35	.78	−.01	−.20	.00	−.16
**Benevolence**	.53	.59	−.21**	−3.14	.02**	2.78
**Universalism**	.63	.49	−.05	−.92	.01	1.01
**Security**	−.24	.64	−.15+	−1.95	.02*	2.33

Note: + p < .10; *p < .05; **p < .01; *M* = Mean, *SD* = Standard Deviation; *Est* = Multi-level Estimate; *t = t value and number in parentheses = degrees of freedom*.

### Temporal changes in perceived value-related differences

The linear and quadratic temporal effects for perceived value-related differences across the pre-, during- and post-phases of the missions are presented in Table 3. There were negative linear and positive quadratic effects for perceived difference related to achievement, power and benevolence indicating that perceived difference scores linked to these values declined and then increased across the phases of the missions. Negative linear trends (p < 0.10) were observed for differences in self-direction, stimulation, hedonism and tradition, suggesting that perceived difference scores associated with these values generally declined over the course of the missions. There was also a trend for a positive quadratic effect for hedonism indicating that after an initial decrease there was an increase in scores for this variable as missions went on. Thus, although these values were generally low in the value hierarchy of the astronauts their within‑mission variability suggests sensitivity to situational and contextual demands.

**Table 3:** Multi-level model results for time-based changes in perceived personal value-related differences.

			Linear		Quadratic	
	*M*	*SD*	*Est*	*t (67)*	*Est*	*t (67)*
**Difference Self-direction**	1.83	.75	−.20+	−1.71	.02	1.34
**Difference Stimulation**	1.87	.82	−.21+	−1.89	.02	1.37
**Difference Hedonism**	1.74	.78	−.21+	−1.75	.02+	1.73
**Difference Achievement**	1.74	.81	−.44**	−3.38	.04**	2.86
**Difference Power**	1.76	.84	−.41**	−2.95	.04**	2.70
**Difference Conformity**	1.72	.87	−.13	−.89	.01	.71
**Difference Tradition**	1.85	1.01	−.22+	−1.78	.02	1.64
Difference Benevolence	1.84	.89	−.31*	−2.33	.03*	2.01

Note: + p < .10; *p < .05; **p < .01; M = Mean, SD = Standard Deviation; Est = Multi-level Estimate; t = t value and number in parentheses = degrees of freedom.

### Temporal changes in perceived value-related tension

Linear and quadratic temporal effects for perceived value-related tenson across the pre-, during- and post-phases of the missions are presented in Table 4. There was a negative linear and positive quadratic effect for perceived value-related tension scores for benevolence suggesting that tension linked to this value declined and then increased across the different phases of the missions. Like findings for perceived value-related differences, there were additional negative linear and positive quadratic trends for stimulation and conformity suggesting that value-related tension scores in these areas were found to initially decline and then increase across the missions.

**Table 4:** Multi-level model results for time-based changes in perceived personal value-related tension.

			Linear		Quadratic	
	*M*	*SD*	*Est*	*t (67)*	*Est*	*t (67)*
**Tension Self-direction**	1.60	.78	−.17	−1.28	.02	1.41
**Tension Stimulation**	1.46	.78	−.26+	−1.96	.02+	1.79
**Tension Hedonism**	1.54	.91	−.14	−.99	.01	.79
**Tension Achievement**	1.59	.93	−.20	−1.31	.02	1.02
**Tension Power**	1.63	.88	−.22	−1.62	.02	1.33
**Tension Conformity**	1.60	.92	−.30+	−1.88	.03+	1.77
**Tension Tradition**	1.74	1.00	−.14	−.97	.01	.98
**Tension Benevolence**	1.47	.80	−.40**	−2.75	.03*	2.34

Note: + p < .10; *p < .05; **p < .01; M = Mean, SD = Standard Deviation; Est = Multi-level Estimate; t = t value and number in parentheses = degrees of freedom.

### Relationships between perceived value-related differences and value-related tension

Findings from the multilevel models testing the relationship between perceived value-related differences and attributions of those differences to value-related tension are presented in Table 5. Significant effects were observed for seven of the eight relationships, where perceived differences in the discrete values of self-direction, hedonism, achievement, power, conformity, tradition, and benevolence were significant positive predictors of tension attributed to those values.

**Table 5:** Multi-level model results for relationship between perceived personal value-related differences and tension.

	*Est*	*t (67)*
**Self-direction differences>> tension**	.46**	4.16
**Stimulation differences>> tension**	.17	1.37
**Hedonism differences>> tension**	.57**	4.86
**Achievement differences>> tension**	.45**	3.87
**Power differences>> tension**	.23*	2.19
**Conformity differences>> tension**	.27*	2.20
**Tradition differences>> tension**	.56**	5.21
**Benevolence differences>> tension**	.38**	3.81

Note: *p < .05; **p < .01; Est = Multi-level Estimate; t = t value and number in parentheses = degrees of freedom.

## Discussion

This study aimed to better understand the dynamics of motivation and associations with interpersonal tension among crew members during long-duration space missions based on unique data from astronauts. Consistent with our expectations, personal values (representing motivational goals) showed high temporal variability. However, in contrast to other research (23), we did not observe substantial differences between pre- and post-mission assessments; the only notable rank-order change was an increased emphasis on power after the mission. The most striking results across the mission was the negative linear and positive quadratic effects observed for benevolence, indicating that astronauts placed decreasing emphasis on benevolence (the wellbeing on others) during the initial half of the mission, with the lowest scores around the midpoint, followed by a subsequent increase. The opposite effects were observed for power and hedonism (an increase focus on pleasure to self), which increased over time before declining into the later phases and post-mission. In line with organizational research, perceptions of value congruence varied over time [54,61]. As expected, perceived value divergence between crew members was strongly and positively associated with intra-crew tension.

### Alterations in personal values

Apart from a heightened emphasis on power post-mission, the personal values scores demonstrated remarkable stability when comparing pre- and post-mission assessments. Overall, astronauts rated their core values, particularly universalism, benevolence, and tradition, as highly concordant. These findings are broadly consistent to profiles of other populations operating in extreme environments [62]. The increase in emphasis on power post-mission suggests that astronauts placed greater importance on social recognition, aligning with results from other studies [23,25]. This observation related to power is also consistent with data from Russian astronauts, obtained through a semantic differential method, indicating a stronger emphasis on dominance and independence following the mission [63]. In explaining this finding, Suedfeld et al. [22,23] suggested that post-flight, astronauts might have been attentive to how their space mission experience could facilitate their future careers and goals. They also noted post-mission increases in transcendence, a construct encompassing spirituality and universality. While this pre-to-post mission difference was not observed in our study, at the group level, universalism consistently ranked at the top of the value hierarchy.

Methodological differences might explain different findings between prior studies and the current work. Whereas the present research assessed personal values using self-report and standardized scales, Suedfeld et al. used text analysis of astronaut narratives that included biographies, public speeches, and media interviews as proxies for values. In the latter research, the time elapsed since returning to Earth might have played a role. The post-mission data in this study was collected shortly after the mission (7–10 days), while the material underlying text analysis could reflect astronauts’ experiences after a long passage of time and relied on retrospective accounts, which may be subject to various types of bias and not fully encapsulate the experience at the time of flight. We suggest that future research should compare these different methodological approaches based on data from the same subjects. Also, to better understand the long-term effect of space mission experiences on personal value priorities, prospective space crew members should be asked to complete the PCVQ for a more extended period post-mission.

Emphasis on benevolence and power were linearly related to the passage of time. These two values have in common that they serve as social motivators related to recognition and social connectedness. The significant decline in benevolence (referring to emphasis on the welfare of those with whom one is frequently in personal contact) is consistent with results from two space simulation studies conducted as part of the Mars500 project [5,6]. When considered in combination with the linear increase in emphasis on power and hedonism, these findings suggest that astronauts may have become more focused on their own self-interests, possibly indicating a decline interpersonal attentiveness with implications for crew cohesion. This interpretation aligns with research on astronaut journals from ISS missions showing a decrease in positive comments about team interactions as the mission progressed [26], and observations that that astronauts tended to depict their fellow crew members in more polarized, “black and white” terms as the mission advanced [37,64]. Findings from space simulation studies are also supportive of this explanation, showing increased crew tension over time [45,58,64–66]. However, notably, a recent study simulating a lunar manned mission observed the opposite pattern, with crew tension escalating early in confinement and then decreasing after being addressed by a psychological specialist on the ground [67].

Findings from the quadratic analyses indicate that the emphasis on benevolence reached its lowest point around the midpoint of the mission. This finding concurs with observations in several studies suggesting that individuals in isolated and confined conditions tend to experience more negative patterns of functioning (e.g., negative mood changes) around halfway or in the third quarter, regardless of the actual duration and the type of environment [68]. Although it might be possible to consider these dynamics through the lens of a so-called “third quarter phenomenon” [68,69], but research evidence is unequivocal [70,71], the observed decline in benevolence coincided with the replacement of three members of the crew and the change in crew Commander. The arrival of new crew members signifies a discrete transition period, and an adjustment in value priorities may have been a biproduct of and even a functional regulatory mechanism for integrating those crew members. The peak emphasis on power in the same phase supports this view and aligns with group formation theory [72], also concurring with observations during space simulation studies [67,73]. When a new team is established, members typically enter a “storming phase” characterized by struggles for dominance, leadership, and acceptance necessary steps toward forming a cohesive and functional team.

A positive quadratic effect for emphasis on security suggests a pattern similar to that of benevolence, where scores first declined and then increased across the different mission phases. According to Schwartz and Bardi [74] security is linked to deficit needs and tend to become more salient when threatened. The observed trajectory aligns with the need for a heightened focus on safety at the beginning and near the end of a space mission, given the increased risks associated with launch and returning to Earth.

### Value diversity as source of tension

While astronauts consistently reported little value-related crew tension, there were strong and positive associations between perceived value incongruence and intra-crew tension, regardless of the specific value involved. These findings align with social psychological theories [49,50] which emphasize the importance of perceived value congruence for attraction and effective team dynamics. Nevertheless, the data does not allow for a definitive conclusion about the direction of causality. It is possible that perceived value incongruence does not directly trigger intra-crew tension, but rather that experiencing tension heightens sensitivity to differences in values. The relationship could be bidirectional or indeed influenced by other factors.

In the case of benevolence, the perceived value incongruence and tension exhibited the same temporal pattern as the individual value scores, a finding aligning with previous research [6]. As astronauts placed less emphasis on benevolence as a core personal value, they might have been less likely to perceive differences in- and tension associated with this value. Conversely, when astronauts place a strong emphasis on caring for others and fostering harmony, they might be more attuned to variations in how individuals express kindness and support, and as such associated tension would be more pronounced.

The observed fluctuations in perceived value congruence are in line with research conducted in earth bound contexts [54]. Interestingly, the temporal variability differed depending on the specific value, a topic that, to our knowledge, has received less attention in the literature. While perception of congruence in benevolence, power and achievement fluctuated throughout the mission (observed as both linear and quadratic effects), the astronauts experienced being more similar in self-direction, stimulation, hedonism and tradition. This may indicate that divergence in these areas became less salient to the astronauts over time, possibly as crew members developed a shared identity and adjusted to the microculture within the team. This interpretation aligns with research indicating that fit perceptions may change in response to socialization [53].

### Methodological limitations

The findings described should be interpreted with recognition of the limitations of this research. As highlighted by Kirrane et al. [43], our measures of value-heterogeneity and tension may not fully capture the complexity of group dynamics. For instance, a scale score for value differences could reflect that all crew members are dissimilar in that value, that subgroups differ from each other, or that one member deviates from the rest. Similarly, reported tension might involve only two astronauts, several subgroups, or the entire crew. We also recognize that “tension” is a broad term that does not specify the severity, acuteness, or whether it is openly expressed. Marcinkowski et al. [75] distinguished between different types of conflicts within isolated and confined teams, noting that tension, defined as one conflict category, often involved subjective attributions and blame. This aligns with our approach in this study, in which we focused on intra-crew tension as perceived and experienced by the astronauts themselves, viewing it through their subjective lens.

Access to psychological data from space is highly restricted, and crew time allocated for specific experiments on the space station can change depending on off-nominal events or high-priority tasks. As a result, there was variability in mission days when the astronauts completed the questionnaire. Obviously, with the small sample size available for this study, any conclusions should be made with caution. Also, the large individual variability in responses should be kept in mind though this is somewhat accounted for in the analytical approach used. The sample consisted exclusively of male astronauts. Empirical evidence from the general population shows that priorities assigned to specific personal values systematically differ across cultures and genders [9]. In astronaut populations, such variability might be reduced due to the rigorous selection procedures and standardized training requirements implemented across agencies. Nonetheless, we encourage further research involving female crewmembers and astronauts from multiple agencies, including emerging space programs such as the Indian Space Research Organisation (ISRO), China National Space Administration (CNSA), and the United Arab Emirates Space Agency (UAESA), whose representatives are increasingly prominent in space mission operations.

As most participants were the sole crew representative, we cannot confidently determine the validity of their subjective perspectives in relation to other members of their crew. Additionally, response biases are always a concern in research relying on self-reports. However, according to Schwartz [19] when respondents are asked to indicate their own values, the impact of social desirability appears to be relatively minor. Nevertheless, despite assurances of confidentiality, crew members might have been hesitant to report tension, fearing that sharing such information could negatively affect their recognition or future career prospects. Response bias may have contributed to the generally low levels of intra-crew tension reported. On the other hand, low tension scores could also reflect effective coping strategies, where crew members manage interpersonal differences before they escalate into overt tension.

## Conclusion

This study is the first to analyze psychometric data on personal values collected from astronauts during actual space missions, offering novel insights into how values alter and impact upon indicators of interpersonal functioning over long-duration ISS stays (4–7 months). A key strength of this study is the ability to make direct comparisons with data from space simulation studies that used the same instruments and data collection schedule. While having an Earth-based matched control group would have been ideal, this is challenging to achieve due to logistical and financial constraints [51]. Nonetheless, comparisons with space simulation studies can help to some extent in addressing that limitation. Our findings confirm previous results from space simulation studies, indicating that crew members’ personal values representing their motivational goals alter over time. One of the most notable findings, consistent with earlier simulation research, is the decline in emphasis on benevolence, and simultaneously, increased focus on power and hedonism, suggesting a shift toward more self-centered priorities. The quadratic trajectories observed for several individual value scores, perceived value differences, and intra-crew tension imply that these dynamics are fluid and subject to change, especially around the mid-mission phase when crew composition was altered.

Understanding these psychological and team processes is vital for successful long-duration space exploration- such as missions to Mars and beyond. Without high motivation and cohesion, collaboration and performance are likely to falter, posing risks to safety. Our research makes a novel contribution and advances the field by demonstrating that crew members modify their motivational goals as the mission progresses. While we cannot determine the underlying reasons for these shifts and whether they have a positive or negative impact on adaptation, we propose that such value changes may reflect both an adaptive response to the context and a component of flexible self-regulation-a key determinant of psychological resilience [33].

In light of the present findings, assessment of interpersonal compatibility in personal values as part of crew assessment, selection and composition endeavors could potentially reduce tensions. There was a relatively high stability in individual value hierarchies when comparing the scores before and after the mission. However, because individual value hierarchies seem to fluctuate during the missions, it is important to provide targeted training and timely in-flight support that enables crew members to effectively cope with issues stemming from perceived value incongruence. Studies from general organizations and other extreme environment contexts suggest coping strategies that could be incorporated into training to help mitigate potential adverse effects [61,76,77]. Additionally, understanding how motivational patterns change over the course of a mission has important implications for the design of support systems in Mission Control. Proactive, value-informed interventions could help maintain an optimal activation, promote resilience and enhance team dynamics like cohesion, ultimately supporting safe and successful long-duration missions.

## Supporting information

S1 File Syntax for statistical analysis. (DOCX)
