# Supplementary material for: Value dynamics and interpersonal tension among astronauts during International Space Station missions
Source: PLoS One. 2026 Jul 8;21(7):e0351965. doi: 10.1371/journal.pone.0351965 (PMC13345256; doi:10.1371/journal.pone.0351965)
Supplement: S1 File — (DOCX) [file pone.0351965.s001.docx]

***Descriptive statistics for personal values***

jaspDescriptives::Descriptives(

version = "0.19.2",

formula = ~ SELFD + STIM + HED + ACH + POW + SEC + CONF + TRAD + BEN + UNI)

***Multi-level models for time-based changes in personal values***

jaspMixedModels::MixedModelsLMM(

version = "0.19.2",

formula = SELFD ~ Linear + Quadratic + (1 | ID),

contrasts = NULL,

fixedEffectEstimate = TRUE,

modelSummary = TRUE,

plotBackgroundData = ~ ID.nominal,

trendsContrasts = NULL)

jaspMixedModels::MixedModelsLMM(

version = "0.19.2",

formula = STIM ~ Linear + Quadratic + (1 | ID),

contrasts = NULL,

fixedEffectEstimate = TRUE,

modelSummary = TRUE,

plotBackgroundData = ~ ID.nominal,

trendsContrasts = NULL)

jaspMixedModels::MixedModelsLMM(

version = "0.19.2",

formula = HED ~ Linear + Quadratic + (1 | ID),

contrasts = NULL,

fixedEffectEstimate = TRUE,

modelSummary = TRUE,

plotBackgroundData = ~ ID.nominal,

trendsContrasts = NULL)

jaspMixedModels::MixedModelsLMM(

version = "0.19.2",

formula = ACH ~ Linear + Quadratic + (1 | ID),

contrasts = NULL,

fixedEffectEstimate = TRUE,

modelSummary = TRUE,

plotBackgroundData = ~ ID.nominal,

trendsContrasts = NULL)

jaspMixedModels::MixedModelsLMM(

version = "0.19.2",

formula = POW ~ Linear + Quadratic + (1 | ID),

contrasts = NULL,

fixedEffectEstimate = TRUE,

modelSummary = TRUE,

plotBackgroundData = ~ ID.nominal,

trendsContrasts = NULL)

jaspMixedModels::MixedModelsLMM(

version = "0.19.2",

formula = CONF ~ Linear + Quadratic + (1 | ID),

contrasts = NULL,

fixedEffectEstimate = TRUE,

modelSummary = TRUE,

plotBackgroundData = ~ ID.nominal,

trendsContrasts = NULL)

jaspMixedModels::MixedModelsLMM(

version = "0.19.2",

formula = TRAD ~ Linear + Quadratic + (1 | ID),

contrasts = NULL,

fixedEffectEstimate = TRUE,

modelSummary = TRUE,

plotBackgroundData = ~ ID.nominal,

trendsContrasts = NULL)

jaspMixedModels::MixedModelsLMM(

version = "0.19.2",

formula = BEN ~ Linear + Quadratic + (1 | ID),

contrasts = NULL,

fixedEffectEstimate = TRUE,

modelSummary = TRUE,

plotBackgroundData = ~ ID.nominal,

trendsContrasts = NULL)

jaspMixedModels::MixedModelsLMM(

version = "0.19.2",

formula = UNI ~ Linear + Quadratic + (1 | ID),

contrasts = NULL,

fixedEffectEstimate = TRUE,

modelSummary = TRUE,

plotBackgroundData = ~ ID.nominal,

trendsContrasts = NULL)

jaspMixedModels::MixedModelsLMM(

version = "0.19.2",

formula = SEC ~ Linear + Quadratic + (1 | ID),

contrasts = NULL,

fixedEffectEstimate = TRUE,

modelSummary = TRUE,

plotBackgroundData = ~ ID.nominal,

trendsContrasts = NULL)

***Descriptive statistics for perceived personal value-related differences***

jaspDescriptives::Descriptives(

version = "0.19.2",

formula = ~ SELFD_DIFF + STIM_DIFF + HED_DIFF + ACH_DIFF + POW_DIFF + CONF_DIFF + TRAD_DIFF + BEN_DIFF)

***Multi-level models for time-based changes in perceived personal value-related differences***

jaspMixedModels::MixedModelsLMM(

version = "0.19.2",

formula = SELFD_DIFF ~ Linear + Quadratic + (1 | ID),

fixedEffectEstimate = TRUE,

modelSummary = TRUE,

plotBackgroundData = ~ ID.nominal)

jaspMixedModels::MixedModelsLMM(

version = "0.19.2",

formula = STIM_DIFF ~ Linear + Quadratic + (1 | ID),

fixedEffectEstimate = TRUE,

modelSummary = TRUE,

plotBackgroundData = ~ ID.nominal)

jaspMixedModels::MixedModelsLMM(

version = "0.19.2",

formula = HED_DIFF ~ Linear + Quadratic + (1 | ID),

fixedEffectEstimate = TRUE,

modelSummary = TRUE,

plotBackgroundData = ~ ID.nominal)

jaspMixedModels::MixedModelsLMM(

version = "0.19.2",

formula = ACH_DIFF ~ Linear + Quadratic + (1 | ID),

fixedEffectEstimate = TRUE,

modelSummary = TRUE,

plotBackgroundData = ~ ID.nominal)

jaspMixedModels::MixedModelsLMM(

version = "0.19.2",

formula = POW_DIFF ~ Linear + Quadratic + (1 | ID),

fixedEffectEstimate = TRUE,

modelSummary = TRUE,

plotBackgroundData = ~ ID.nominal)

jaspMixedModels::MixedModelsLMM(

version = "0.19.2",

formula = CONF_DIFF ~ Linear + Quadratic + (1 | ID),

fixedEffectEstimate = TRUE,

modelSummary = TRUE,

plotBackgroundData = ~ ID.nominal)

jaspMixedModels::MixedModelsLMM(

version = "0.19.2",

formula = TRAD_DIFF ~ Linear + Quadratic + (1 | ID),

fixedEffectEstimate = TRUE,

modelSummary = TRUE,

plotBackgroundData = ~ ID.nominal)

jaspMixedModels::MixedModelsLMM(

version = "0.19.2",

formula = BEN_DIFF ~ Linear + Quadratic + (1 | ID),

fixedEffectEstimate = TRUE,

modelSummary = TRUE,

plotBackgroundData = ~ ID.nominal)

***Descriptive statistics for perceived personal value-related tension***

jaspDescriptives::Descriptives(

version = "0.19.2",

formula = ~ SELFD_TENSE + STIM_TENSE + HED_TENSE + ACH_TENSE + POW_TENSE + CONF_TENSE + TRAD_TENSE + BEN_TENSE)

***Multi-level models for time-based changes in perceived personal value-related tension***

jaspMixedModels::MixedModelsLMM(

version = "0.19.2",

formula = SELFD_TENSE ~ Linear + Quadratic + (1 | ID),

fixedEffectEstimate = TRUE,

modelSummary = TRUE,

plotBackgroundData = ~ ID.nominal)

jaspMixedModels::MixedModelsLMM(

version = "0.19.2",

formula = STIM_TENSE ~ Linear + Quadratic + (1 | ID),

fixedEffectEstimate = TRUE,

modelSummary = TRUE,

plotBackgroundData = ~ ID.nominal)

jaspMixedModels::MixedModelsLMM(

version = "0.19.2",

formula = HED_TENSE ~ Linear + Quadratic + (1 | ID),

fixedEffectEstimate = TRUE,

modelSummary = TRUE,

plotBackgroundData = ~ ID.nominal)

jaspMixedModels::MixedModelsLMM(

version = "0.19.2",

formula = ACH_TENSE ~ Linear + Quadratic + (1 | ID),

fixedEffectEstimate = TRUE,

modelSummary = TRUE,

plotBackgroundData = ~ ID.nominal)

jaspMixedModels::MixedModelsLMM(

version = "0.19.2",

formula = POW_TENSE ~ Linear + Quadratic + (1 | ID),

fixedEffectEstimate = TRUE,

modelSummary = TRUE,

plotBackgroundData = ~ ID.nominal)

jaspMixedModels::MixedModelsLMM(

version = "0.19.2",

formula = CONF_TENSE ~ Linear + Quadratic + (1 | ID),

fixedEffectEstimate = TRUE,

modelSummary = TRUE,

plotBackgroundData = ~ ID.nominal)

jaspMixedModels::MixedModelsLMM(

version = "0.19.2",

formula = TRAD_TENSE ~ Linear + Quadratic + (1 | ID),

fixedEffectEstimate = TRUE,

modelSummary = TRUE,

plotBackgroundData = ~ ID.nominal)

jaspMixedModels::MixedModelsLMM(

version = "0.19.2",

formula = BEN_TENSE ~ Linear + Quadratic + (1 | ID),

fixedEffectEstimate = TRUE,

modelSummary = TRUE,

plotBackgroundData = ~ ID.nominal)

***Multi-level models for relationship between perceived personal value-related differences and tension***

jaspMixedModels::MixedModelsLMM(

version = "0.19.2",

formula = SELFD_TENSE ~ SELFD_DIFF + (1 | ID),

contrasts = NULL,

fixedEffectEstimate = TRUE,

modelSummary = TRUE,

plotBackgroundData = ~ ID.nominal,

trendsContrasts = NULL)

jaspMixedModels::MixedModelsLMM(

version = "0.19.2",

formula = STIM_TENSE ~ STIM_DIFF + (1 | ID),

contrasts = NULL,

fixedEffectEstimate = TRUE,

modelSummary = TRUE,

plotBackgroundData = ~ ID.nominal,

trendsContrasts = NULL)

jaspMixedModels::MixedModelsLMM(

version = "0.19.2",

formula = HED_TENSE ~ HED_DIFF + (1 | ID),

contrasts = NULL,

fixedEffectEstimate = TRUE,

modelSummary = TRUE,

plotBackgroundData = ~ ID.nominal,

trendsContrasts = NULL)

jaspMixedModels::MixedModelsLMM(

version = "0.19.2",

formula = ACH_TENSE ~ ACH_DIFF + (1 | ID),

contrasts = NULL,

fixedEffectEstimate = TRUE,

modelSummary = TRUE,

plotBackgroundData = ~ ID.nominal,

trendsContrasts = NULL)

jaspMixedModels::MixedModelsLMM(

version = "0.19.2",

formula = POW_TENSE ~ POW_DIFF + (1 | ID),

contrasts = NULL,

fixedEffectEstimate = TRUE,

modelSummary = TRUE,

plotBackgroundData = ~ ID.nominal,

trendsContrasts = NULL)

jaspMixedModels::MixedModelsLMM(

version = "0.19.2",

formula = CONF_TENSE ~ CONF_DIFF + (1 | ID),

contrasts = NULL,

fixedEffectEstimate = TRUE,

modelSummary = TRUE,

plotBackgroundData = ~ ID.nominal,

trendsContrasts = NULL)

jaspMixedModels::MixedModelsLMM(

version = "0.19.2",

formula = TRAD_TENSE ~ TRAD_DIFF + (1 | ID),

contrasts = NULL,

fixedEffectEstimate = TRUE,

modelSummary = TRUE,

plotBackgroundData = ~ ID.nominal,

trendsContrasts = NULL)

jaspMixedModels::MixedModelsLMM(

version = "0.19.2",

formula = BEN_TENSE ~ BEN_DIFF + (1 | ID),

contrasts = NULL,

fixedEffectEstimate = TRUE,

modelSummary = TRUE,

plotBackgroundData = ~ ID.nominal,

trendsContrasts = NULL)

***Figures for time-based changes in personal values***

jaspDescriptives::flexplot(

version = "0.19.2",

formula = SELFD ~ Linear,

jitx = 0,

nameX = "Linear",

nameY = "SELFD")

jaspDescriptives::flexplot(

version = "0.19.2",

formula = STIM ~ Linear,

jitx = 0,

nameX = "Linear",

nameY = "STIM")

jaspDescriptives::flexplot(

version = "0.19.2",

formula = HED ~ Linear,

jitx = 0,

nameX = "Linear",

nameY = "HED")

jaspDescriptives::flexplot(

version = "0.19.2",

formula = ACH ~ Linear,

jitx = 0,

nameX = "Linear",

nameY = "ACH")

jaspDescriptives::flexplot(

version = "0.19.2",

formula = POW ~ Linear,

jitx = 0,

nameX = "Linear",

nameY = "POW")

jaspDescriptives::flexplot(

version = "0.19.2",

formula = CONF ~ Linear,

jitx = 0,

nameX = "Linear",

nameY = "CONF")

jaspDescriptives::flexplot(

version = "0.19.2",

formula = TRAD ~ Linear,

jitx = 0,

nameX = "Linear",

nameY = "TRAD")

jaspDescriptives::flexplot(

version = "0.19.2",

formula = BEN ~ Linear,

jitx = 0,

nameX = "Linear",

nameY = "BEN")

jaspDescriptives::flexplot(

version = "0.19.2",

formula = UNI ~ Linear,

jitx = 0,

nameX = "Linear",

nameY = "UNI")

jaspDescriptives::flexplot(

version = "0.19.2",

formula = SEC ~ Linear,

jitx = 0,

nameX = "Linear",

nameY = "SEC")

***Figures for time-based changes in perceived personal value-related differences***

jaspDescriptives::flexplot(

version = "0.19.2",

formula = SELFD_DIFF ~ Linear,

jitx = 0,

nameX = "Linear",

nameY = "SELFD_DIFF")

jaspDescriptives::flexplot(

version = "0.19.2",

formula = STIM_DIFF ~ Linear,

jitx = 0,

nameX = "Linear",

nameY = "STIM_DIFF")

jaspDescriptives::flexplot(

version = "0.19.2",

formula = HED_DIFF ~ Linear,

jitx = 0,

nameX = "Linear",

nameY = "HED_DIFF")

jaspDescriptives::flexplot(

version = "0.19.2",

formula = ACH_DIFF ~ Linear,

jitx = 0,

nameX = "Linear",

nameY = "ACH_DIFF")

jaspDescriptives::flexplot(

version = "0.19.2",

formula = POW_DIFF ~ Linear,

jitx = 0,

nameX = "Linear",

nameY = "POW_DIFF")

jaspDescriptives::flexplot(

version = "0.19.2",

formula = CONF_DIFF ~ Linear,

jitx = 0,

nameX = "Linear",

nameY = "CONF_DIFF")

jaspDescriptives::flexplot(

version = "0.19.2",

formula = TRAD_DIFF ~ Linear,

jitx = 0,

nameX = "Linear",

nameY = "TRAD_DIFF")

jaspDescriptives::flexplot(

version = "0.19.2",

formula = BEN_DIFF ~ Linear,

jitx = 0,

nameX = "Linear",

nameY = "BEN_DIFF")

***Figures for time-based changes in perceived personal value-related tension***

jaspDescriptives::flexplot(

version = "0.19.2",

formula = SELFD_TENSE ~ Linear,

jitx = 0,

nameX = "Linear",

nameY = "SELFD_TENSE")

jaspDescriptives::flexplot(

version = "0.19.2",

formula = STIM_TENSE ~ Linear,

jitx = 0,

nameX = "Linear",

nameY = "STIM_TENSE")

jaspDescriptives::flexplot(

version = "0.19.2",

formula = HED_TENSE ~ Linear,

jitx = 0,

nameX = "Linear",

nameY = "HED_TENSE")

jaspDescriptives::flexplot(

version = "0.19.2",

formula = ACH_TENSE ~ Linear,

jitx = 0,

nameX = "Linear",

nameY = "ACH_TENSE")

jaspDescriptives::flexplot(

version = "0.19.2",

formula = POW_TENSE ~ Linear,

jitx = 0,

nameX = "Linear",

nameY = "POW_TENSE")

jaspDescriptives::flexplot(

version = "0.19.2",

formula = CONF_TENSE ~ Linear,

jitx = 0,

nameX = "Linear",

nameY = "CONF_TENSE")

jaspDescriptives::flexplot(

version = "0.19.2",

formula = TRAD_TENSE ~ Linear,

jitx = 0,

nameX = "Linear",

nameY = "TRAD_TENSE")

jaspDescriptives::flexplot(

version = "0.19.2",

formula = BEN_TENSE ~ Linear,

jitx = 0,

nameX = "Linear",

nameY = "BEN_TENSE")
